# Scalp HFO rates decrease after successful epilepsy surgery and are not impacted by the skull defect resulting from craniotomy

**DOI:** 10.1038/s41598-022-05373-x

**Published:** 2022-01-25

**Authors:** Dorottya Cserpan, Antonio Gennari, Luca Gaito, Santo Pietro Lo Biundo, Ruth Tuura, Johannes Sarnthein, Georgia Ramantani

**Affiliations:** 1 Department of Neuropediatrics, University Children’s Hospital Zurich, Steinwiesstrasse 75, 8032 Zurich, Switzerland; 2 MR-Research Centre, University Children’s Hospital Zurich, Zurich, Switzerland; 3 Department of Neurosurgery, University Hospital Zurich, Zurich, Switzerland; 4 University of Zurich, Zurich, Switzerland; 5 Children’s Research Centre, University Children’s Hospital Zurich, Zurich, Switzerland; 6 Klinisches Neurozentrum Zürich, University Hospital Zurich, Zurich, Switzerland

**Keywords:** Diagnostic markers, Paediatric research, Epilepsy

## Abstract

Epilepsy surgery can achieve seizure freedom in selected pediatric candidates, but reliable postsurgical predictors of seizure freedom are missing. High frequency oscillations (HFO) in scalp EEG are a new and promising biomarker of treatment response. However, it is unclear if the skull defect resulting from craniotomy interferes with HFO detection in postsurgical recordings. We considered 14 children with focal lesional epilepsy who underwent presurgical evaluation, epilepsy surgery, and postsurgical follow-up of ≥ 1 year. We identified the nearest EEG electrodes to the skull defect in the postsurgical MRI. We applied a previously validated automated HFO detector to determine HFO rates in presurgical and postsurgical EEG. Overall, HFO rates showed a positive correlation with seizure frequency (*p* < 0.001). HFO rates in channels over the HFO area decreased following successful epilepsy surgery, irrespective of their proximity to the skull defect (*p* = 0.005). HFO rates in channels outside the HFO area but near the skull defect showed no increase following surgery (*p* = 0.091) and did not differ from their contralateral channels (*p* = 0.726). Our observations show that the skull defect does not interfere with postsurgical HFO detection. This supports the notion that scalp HFO can predict postsurgical seizure freedom and thus guide therapy management in focal lesional epilepsy.

## Introduction

For children with drug-resistant focal lesional epilepsy, epilepsy surgery is the only treatment that bears the potential of cure, as recently shown in a randomized controlled trial^1^. Overall, seizure freedom is attained in two-thirds of patients, and cure, defined as seizure freedom off anti-seizure medication (ASM), is achieved in one-third^2–8^. Following surgery, the lack of residual lesions in postsurgical MRI and the lack of epileptic discharges in postsurgical EEG have been suggested as potential predictors of seizure freedom in pediatric cohorts^2–6^, albeit with contradictory results between studies. Considering that pediatric lesional epilepsies are not necessarily MRI-positive^3,5,6^ and that the relationship of the lesion with the epileptogenic zone is not necessarily straightforward^9^, EEG biomarkers appear more suitable for the measurement of residual epileptogenicity and, thus, for the prediction of seizure outcome. Predicting seizure freedom following surgery with confidence is crucial for therapy management and specifically for the timely initiation of ASM withdrawal. Early ASM withdrawal after successful epilepsy surgery determines postsurgical cognitive gains^10,11^, as the only modifiable postsurgical predictor of optimal cognitive outcome^12^.

High frequency oscillations (HFO) in scalp EEG are a new and promising non-invasive epilepsy biomarker providing added prognostic value, particularly in the pediatric population^13^. Originally, scalp HFO have been associated with the seizure onset zone in drug-resistant focal epilepsy^14–21^, thus delineating surgical resection. Currently, scalp HFO are investigated as biomarkers of seizure propensity, disease severity, and treatment response^13^. In our recent study, scalp HFO rates in pediatric drug-resistant focal lesional epilepsy showed a positive correlation with seizure frequency and decreased with seizure cessation following successful epilepsy surgery^14^. This observation underlined the potential of scalp HFO as a widely accessible non-invasive biomarker of treatment response, potentially facilitating outcome prognostication. However, comparisons between consecutive scalp EEG recordings before and after epilepsy surgery are only valid under the assumption that the craniotomy with the resulting skull defect has no significant impact on scalp HFO detection.

In scalp EEG, the major resistive element is the bone with a resistance of around 40,000 Ω for 1 cm^2^ of skull, in contrast to 12,000 Ω for 1 cm^2^ of dura mater, and 1000 Ω for 1 cm^2^ of scalp in adults. EEG signals passing through the intact skull are subject to the averaging and smoothing effects of the bone^22,23^ that skull defects due to craniotomy can partially abolish^24,25^. Signal attenuation and smoothing with bone present and signal accentuation and deblurring with bone absent are both frequency-dependent, i.e., the faster the EEG rhythm, the more pronounced the impact on the signal^24,25^. Therefore, EEG recorded over a skull defect is expected to show higher overall spectral power and a greater proportion of fast components due to decreased high frequency attenuation compared to EEG from a homologous head region with an intact skull, corresponding to a “breach rhythm”^26^. The question remains whether a skull defect following epilepsy surgery can interfere with the detection and interpretation of scalp EEG signals in the HFO range and thus with the validity of scalp HFO as a biomarker of treatment response.

To assess the effect of skull defects resulting from craniotomy on the validity of postsurgical scalp HFO as a biomarker of treatment response, we retrospectively analyzed scalp EEG from children and adolescents with drug-resistant focal lesional epilepsy before and after resective surgery that involved craniotomy. To address the hypothesis that scalp HFO rates decrease over the HFO area following successful epilepsy surgery, irrespective of the distance to the skull defect resulting from craniotomy, we investigated pre- to postsurgical changes in HFO rates depending on the postsurgical seizure outcome and the distance to the skull defect. To address the hypothesis that scalp HFO rates outside the HFO area but close to the skull defect resulting from craniotomy show no increase following surgery, we investigated pre- to postsurgical changes in scalp HFO rates depending on the distance from the skull defect.

## Methods

### Patient recruitment

We prospectively enrolled 37 consecutive children and adolescents with drug-resistant focal lesional epilepsy who underwent presurgical evaluation, resective epilepsy surgery between July 2017 and November 2020, and postsurgical follow-up at the University Children’s Hospital Zurich, according to our institutional protocol. For the current study focusing on the effect of the skull defect resulting from craniotomy on scalp HFO rates, we considered those patients for further analysis that fulfilled the following inclusion criteria: (1) presurgical and postsurgical scalp EEG recorded at a high sampling frequency (> 1000 Hz), (2) containing at least 10 min of NREM sleep, and (3) recorded at > 2 h from the most recent seizure, and (4) follow-up ≥ 1 year after epilepsy surgery.

Presurgical evaluation at ≤ 3 months before surgery included scalp EEG and high-resolution brain MRI, while postsurgical evaluation at three months after surgery included scalp EEG and MRI according to the same protocol. Epilepsy substrates were determined by histopathology. Postsurgical seizure outcome was depicted according to the Engel scale^27^. Seizure frequency was assessed by long-term video-EEG or seizure diaries at the time of the presurgical and postsurgical scalp EEG.

The collection of patient data and the scientific analysis were approved by and performed according to the guidelines and regulations of the local ethics committee (Kantonale Ethikkommission Zürich, KEK-ZH PB-2021–01246). All parents and patients, where applicable, have given written informed consent.

### Electrode positions in relation to the skull defect resulting from craniotomy

Postsurgical MRI scans were performed on a 3-Tesla scanner (Discovery 750®, General Electric Medical System, Milwaukee, WI, USA) with an 8-channel head coil, including a volumetric, whole-brain, T1-weighted sequence (3D-FSPGR-BRAVO sequence; matrix size, 512 × 512 with 300 contiguous slices; field of view: 256 mm; isotropic resolution, 1 mm; sagittal slice orientation; repetition time: 12.1 ms; echo time: 5.2 ms; flip angle: 12°; inversion time: 450 ms). The 3D T1-weighted DICOM images were converted to nifti files using dcm2nii and then loaded into the 3D Slicer (version 4.10). The craniotomy site was identified by an experienced radiologist (A.G.), and the bone flap was manually segmented, using post-processing tools (e.g., windowing and magnification) and switching between image plane orientations. The following smoothing filters were then applied to the generated files: (1) closing, which filled sharp corners and holes in the segmentation, (2) opening, which removed extrusion, and (3) joint smoothly, which evened the segmentation. The resulting images were re-evaluated and adjusted. Finally, the bone flap volumes were calculated, approximating the segmentation areas.

Native nifti files were reloaded into the Geodesic Slicer module 3D Slicer. Geodesic Slicer enables the creation of a triangular mesh morphed on the patient's head, reconstructed from the T1-weighted images. The mesh is modeled according to the geodesic distance measurement, defined as the shortest path between two points in a curved space. After placing four reference points (fiducials) on the nasion, the inion, and the left and right tragi (i.e., anatomical landmarks), the electrode locations of the 10–20 EEG system were automatically generated. The bone flap segmentations were loaded into 3D Slicer, and the distance between the skull defect resulting from craniotomy (skull incision) and the closest electrodes was automatically calculated using classical Euclidean distance formulas for all segmentation borders. For visualization, we used the FieldTrip package (Matlab R2020a).

### Scalp EEG recording and data selection

Patients underwent afternoon nap (N = 18) or whole-night video-EEG (N = 10) with 21 electrodes placed according to the international 10–20 system. Impedances were typically ≤ 5 kΩ. Recordings were performed at a 1024 Hz sampling rate by the Deltamed® (Paris, France) EEG system for afternoon nap recordings or the Micromed® (Mogliano Veneto, Treviso, Italy) EEG system for whole night recordings. Sleep stages were marked by experienced neurophysiologists according to the American Academy of Sleep Medicine (AASM) criteria^28^. We considered the NREM sleep stage N3 for further analysis.

We divided the selected data into 5-min intervals for further processing. During visual pre-processing, 5-min EEG intervals heavily contaminated by artifacts and channels with continuous interference were fully excluded from further analysis. Other artifacts were detected and excluded during the automated HFO detection.

HFO detection and analysis were performed blinded to clinical features of the patients, and the results from HFO analysis were not considered for clinical decision making.

### Automated scalp HFO detection in EEG

To capture the HFO activity with the highest possible spatial resolution, we re-referenced to a bipolar montage using all combinations of neighboring electrodes, thus obtaining 52 bipolar channels^29^.

Scalp HFO detection was conducted with a clinically validated, automated HFO detector^14,29–32^ that operates in three stages^32^. The detector uses Stockwell’s algorithm for the time–frequency transform of the EEG signal^30^. In Stage I, *baseline detection*, a baseline amplitude threshold is determined in artifact-free intervals, selecting epochs of high Stockwell entropy in the ripple band (80–250 Hz). Events exceeding this threshold are marked as events of interest (EoI). In Stage II, *HFO validation by Stockwell transform*, the detector selects those EoI where a high-frequency peak is isolated from the low-frequency activity and marks them as HFO. This step reflects the assumption that HFO are brief events with a distinct high-frequency contribution that stands out from the baseline^30^. Finally, in Stage III, *artifact rejection*, the detector screens EoI selected in stage II to further eliminate artifacts. This step is guided by the assumption that HFO are generated by highly localized cortical tissue and should thus not be detectable across hemispheres^33^. Thus, the detector rejects all EoI co-occurring in homologous channels of the two hemispheres, in addition to those with peak-to-peak amplitude ≥ 40 µV or signal-to-noise ratio < 9. In addition, a z-score is calculated from the 250–500 Hz band-pass-filtered data over time, and events that occur at high z-score timepoints, exceeding the median z-score by 1.5 times the interquartile range for each patient, are rejected. An exemplary HFO event is shown in Supplementary Fig. S1.

The HFO detector was thus applied to each bipolar channel within each selected 5-min data interval. We calculated the HFO rate for each bipolar channel by dividing the number of detected HFO on each channel by the duration of the analyzed EEG recording, resulting in the unit HFO/min.

We controlled for the clinical plausibility of scalp HFO rate distributions by comparing the HFO area with the localization of the presumed epileptogenic zone, as defined by presurgical evaluation, and with the localization of the consequent resection.

There was neither visual marking nor manual rejection of artifacts, rendering the HFO detection fully automated and prospective.

### Channels of interest

*HFO area channels* were defined by consistently high HFO rates across scalp EEG channels for each patient^31^. First, we calculated the rate threshold (97.5^th^ percentile of the HFO rate distribution) from the presurgical EEG across data intervals and channels. Then, we counted the number of intervals with an above-threshold HFO rate for each channel. Finally, we calculated the 97.5^th^ percentile of these occurrence values. Channels with higher occurrence values constituted the *HFO area*.

**Figure 1:**
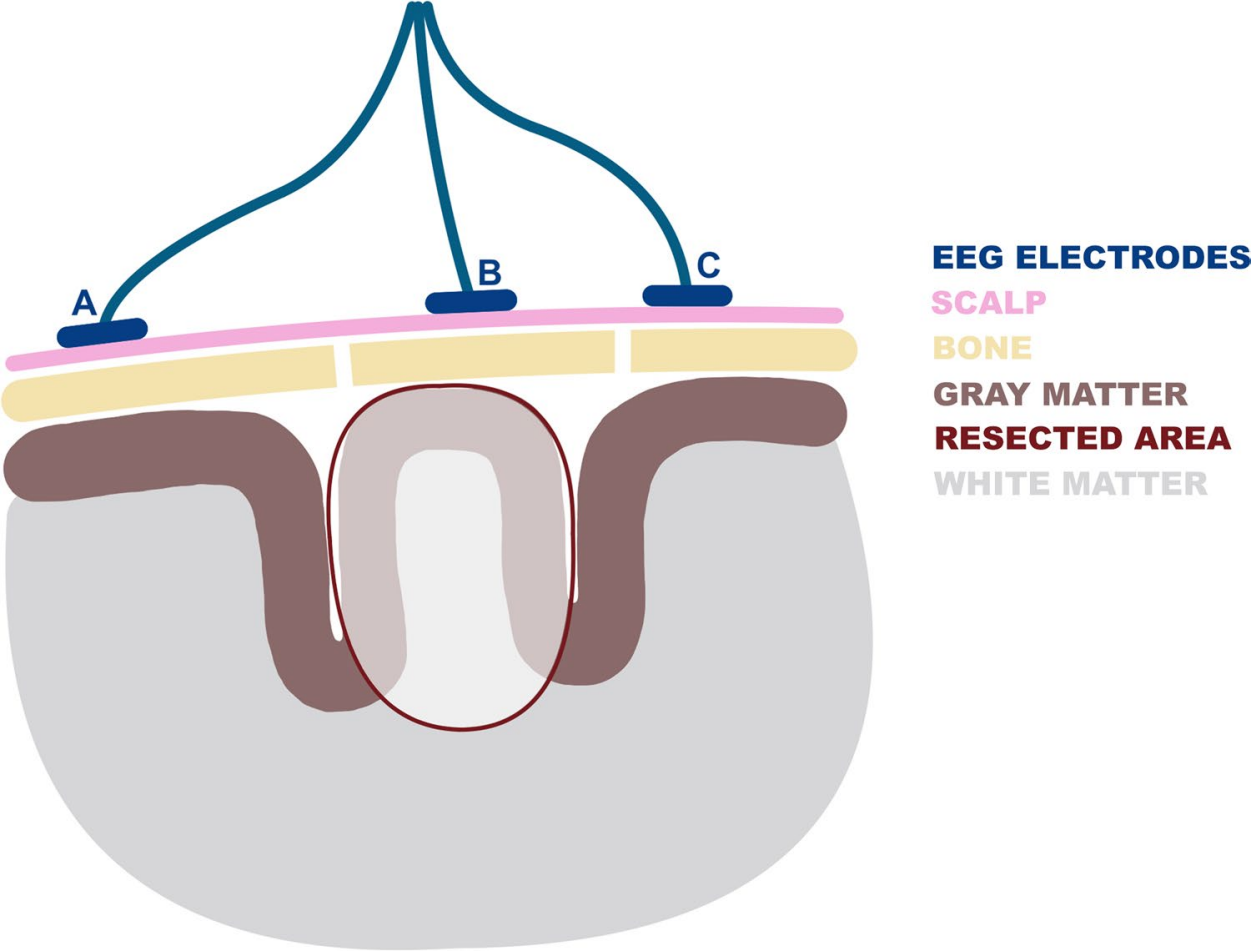
Scalp EEG recording setup following epilepsy surgery. Position of the 10–20 system EEG electrodes following epilepsy surgery in relation to the skull defect resulting from craniotomy and the resected area. For illustrative purposes, we consider that the presurgically defined HFO area overlaps with the resected area depicted here. Electrodes A, B, and C are all located ipsilateral to the craniotomy. Electrode A is located > 1 cm from the skull defect, the HFO area, and the resection area, and thus does not correspond to a channel of interest. Electrode B is located within the HFO area and is thus involved in *HFO area channels*. Electrode C is located outside the HFO and the resection area but close to the skull defect (≤ 1 cm) and is thus involved in *skull defect channels*.

*Skull defect channels* were defined as channels including electrodes located at ≤ 1 cm distance from the skull defect resulting from craniotomy (skull defect electrodes, Fig. 1). For example, assuming that the C3 electrode was located at a distance of 0.3 cm from the skull defect, the channels F3-C3, C3-P3, T5-C3, C3-Pz, C3-Fz would be considered skull defect channels.

*Contralateral skull defect channels* were those contralateral to skull defect channels.

### Statistics

To evaluate scalp HFO rates as an epilepsy biomarker, we compared the HFO rates within the HFO area with seizure frequency as a measure of disease severity^14^.

We then established a classification threshold using the optimal operating point of the receiver operating characteristic (ROC) curve, as in our previous work^14^. In addition, we challenged the predictive performance of the HFO threshold rate methodology by applying two-fold cross-validation. We randomly shuffled the data array and split it into two equal size datasets d_0_ and d_1_, each containing 14 scalp EEG recordings. We then trained on d_0_ and validated on d_1_, followed by training on d_1_ and validating on d_0_, then repeating this procedure two times the number of recordings. We defined as true positive (TP) a scalp EEG showing equal or above-threshold HFO rates in patients with active epilepsy (all patients before surgery, patients with Engel II-IV outcome after surgery). We defined as false positive (FP) a scalp EEG showing equal or above-threshold HFO rates in seizure-free patients after surgery (Engel I). We defined as false negative (FN) a scalp EEG showing below-threshold HFO rates in patients with seizure recurrence after surgery (Engel II-IV). We defined as true negative (TN) a scalp EEG showing below-threshold HFO rates in seizure-free patients after surgery (Engel I). The positive predictive value (PPV) of scalp HFO rates for active epilepsy was calculated as PPV = TP/(TP + FP), the negative predictive value (NPV) as NPV = TN/(TN + FN), and the accuracy = (TP + TN)/N, with N signifying the total number of analyzed EEG recordings.

To investigate the impact of the skull defect on HFO detection, we compared HFO rates and amplitudes averaged over the channels of interest and described distributions by their median and interquartile range (iqr). To characterize the effect of the skull defect resulting from craniotomy on HFO rates, we used the non-parametric Wilcoxon signed-rank test for comparisons of these values in presurgical to postsurgical EEG or between ipsilateral and contralateral channels in the postsurgical EEG for each patient. We used the Spearman's rank correlation to assess the relationship between the seizure frequency and the mean HFO rates within the HFO area.

Statistical analysis was performed with Matlab R2020a. It should be noted that the MATLAB signed-rank function applied in our study handles zero-inflation by considering only non-zero values. Significance was established at *p* < 0.05. We did not perform adjustment for multiple comparisons.

## Results

### Patient characteristics and HFO rates

We included 14 patients (10 female) with mean age 8.9 years at surgery (range 1.5–16.5 years) that fulfilled all inclusion criteria. Etiology included cortical malformations and benign tumors in six patients each, cavernomas in two patients, and one patient with Sturge Weber syndrome. The lateralization of the epileptogenic zone and surgical resection was left in eight patients, and the localization was frontal in six patients, temporal in three, parietal in two, and temporo-parieto-occipital in the remaining three patients. Ten of 14 (71%) patients were seizure-free (Engel Ia) at the last follow-up (mean 2.2 years, range 1.0–4.8 years) following epilepsy surgery, whereas six were seizure-free off ASM (four at ≥ 2-year follow-up). Overall, the median seizure frequency dropped from 20 seizures/month (range 20–1290) at the presurgical EEG to 0 seizures/month (range 0–240) at the postsurgical EEG.

We analyzed 750 min of presurgical and postsurgical EEG data, including 120 min of N2 and 630 min of N3 sleep. In total, we detected 2200 HFO, including 563 in N2 and 1637 in N3 sleep. The median duration of analyzed data per patient was 50 min (iqr 30) with a median of 85 (iqr 168) detected HFO per patient. The localization of the *HFO area* matched on a lobar level the epileptogenic zone, as determined by the electroclinical correlations and the presence of focal MRI lesions in all patients. Following resective surgery through craniotomy, five patients each had one or two skull defect electrodes, one patient had three skull defect electrodes, and three patients had no skull defect electrodes.

The clinical features of our patients, including the location of the skull defect electrodes, are given in Table 1.

**Table 1:** Clinical features of our patients.

Pat. Nr	Sex	Etiology	Age at surgery, years	Resected area	Skull defect electrodes	Follow-up duration, years	Seizure outcome (Engel)	ASM withdrawn
1	f	Ganglioglioma	1.5	R temporal	T4	1.5	IV	No
2	f	Low-grade glioma	2.5	L frontal	None	1.7	Ia	No
3	f	FCD 2b	2.8	R frontal	Fz	1.7	IV	No
4	f	MOGHE	3.6	L frontal	Fz, F3, C3	1.6	Ia	No
5	f	Sturge Weber syndrome	3.8	R temporo-occipital	O2, P4	4.2	Ia	No
6	f	FCD 2a	5.3	R frontal	C4	1.3	IV	No
7	m	FCD 1a	7.8	L temporο-parieto-occipital	T3, F7	4.8	Ia	No
8	m	Ganglioglioma	10.5	L temporal	T5	1.8	Ia	Yes
9	f	Angiocentric glioma	11.4	R parietal	Oz, Pz	1.0	Ia	Yes
10	m	FCD IIa	13.0	L parietal	T3, F7	1.0	IV	No
11	m	Cavernoma	13.1	L frontal	None	2.8	Ia	Yes
12	f	Ganglioglioma	16.0	L temporal	T3, F7	2.7	Ia	Yes
13	f	Cavernoma	16.3	L frontal	None	2.5	Ia	Yes
14	f	FCD IIIb, ganglioglioma	16.5	R temporo-parietal	T6	2.0	Ia	Yes

Clinical features include the etiological substrate, as verified by histopathology, the lobar localization and lateralization of the resected area, the skull defect electrodes in postsurgical EEG, the postsurgical seizure outcome, and the ASM status at last follow-up for each patient.

*Pat.* Patient, *nr* number, *m* male, *f* female, *FCD* focal cortical dysplasia, *MOGHE* mild malformation of cortical development with oligodendroglial hyperplasia and epilepsy, *L* left, *R* right, *ASM* anti-seizure medication.

### HFO rates over the HFO area decrease after successful epilepsy surgery

**Figure 2:**
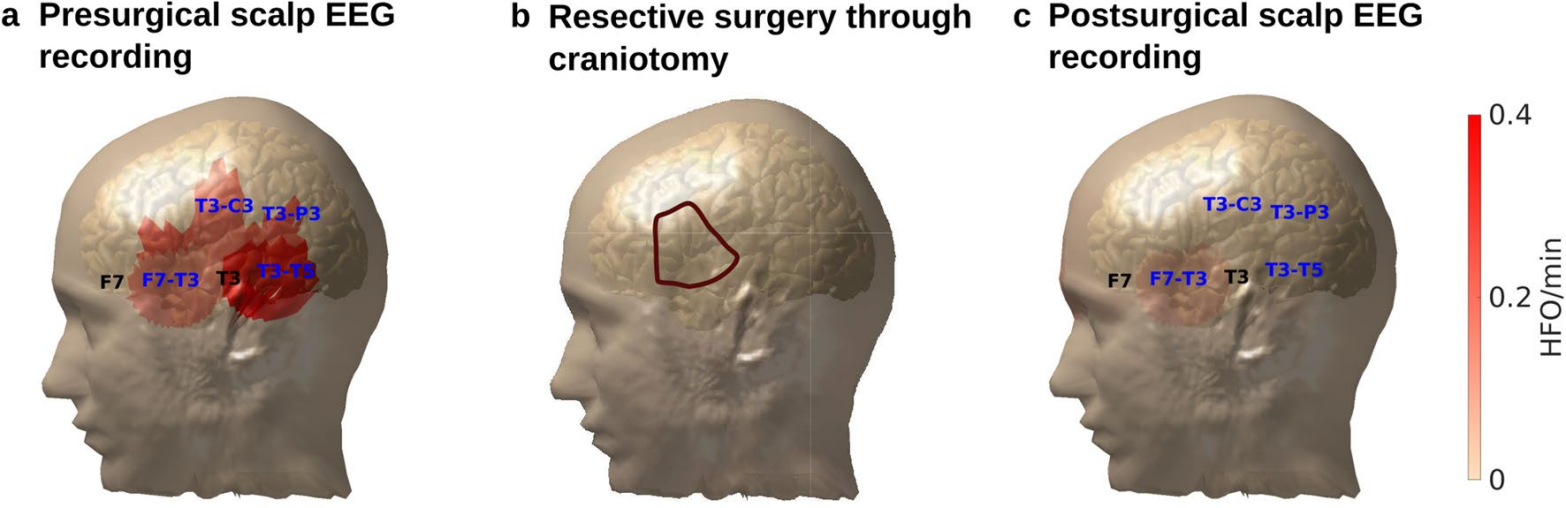
Scalp HFO rate distribution in presurgical and postsurgical EEG in relation to the skull defect resulting from craniotomy. 3D reconstruction of the skull and the cortical surface generated using the presurgical and postsurgical MRI of patient 12, who had a left temporal ganglioglioma. The spatial distribution of HFO rates in presurgical and postsurgical EEG is depicted using areas marked with different shades of red. Different color intensity corresponds to different HFO rates. *HFO area channels* are shown in blue, skull defect electrodes in black**. **(**a**) In the presurgical scalp EEG, high HFO rates were measured in left temporal channels, matching the location of the HFO area, the epileptogenic zone, and the lesion in the anterior portion of the fusiform gyrus. (**b**) Given the lesion location, a pterional craniotomy approach (outlined in brown) was chosen to fully resect the epileptogenic zone and lesion. (**c**) In the postsurgical EEG, HFO rates drastically decreased, in line with postsurgical seizure freedom.

In our cohort, scalp HFO rates in *HFO area channels* correlated with seizure frequency (Spearman’s rho 0.740, *p* < 0.001). Scalp HFO rates in *HFO area channels* exceeded the threshold of 0.124 HFO/min based on the ROC curve (Supplementary Fig. S2a) in 17 EEG recordings, all from patients with active epilepsy (PPV = 100%) and fell below this threshold in 11 recordings, ten from patients that achieved postsurgical seizure freedom (NPV = 91%, accuracy = 96%). The two-fold cross-validation supported the validity of our findings (median PPV = 100%, iqr 0%; median NPV = 83%, iqr 29%; median accuracy = 93%, iqr 7%), while the median HFO rate threshold matched the threshold deriving from the full dataset (median 0.124, iqr 0.001: Supplementary Fig. S2b). Scalp HFO rates over the HFO area decreased following successful epilepsy surgery, as illustrated in Fig. 2 for the exemplary case of patient 12.

**Figure 3:**
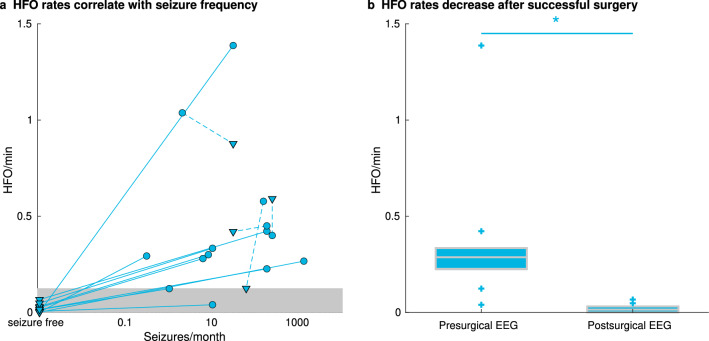
Scalp HFO rates correlate with seizure frequency and decrease after successful epilepsy surgery. (**a**) Scalp HFO rates in *HFO area channels* correlate with seizure frequency (Spearman’s rho 0.740, *p* < 0.001). Presurgical HFO rates are indicated by circles and postsurgical HFO rates by triangles. Pre- and postsurgical HFO rates are connected by a solid line for the patients for whom postsurgical seizure freedom was achieved (Engel I) and by a dotted line for patients for whom postsurgical seizure freedom was not achieved (Engel II–IV). The gray area on the bottom of the graph represents the HFO range corresponding to seizure freedom, based on the 0.124 HFO/min threshold, as established by the ROC curve. 17 of 28 scalp EEG recordings were classified correctly as true positive, ten as true negative, and only one was a false negative. (**b**) Median scalp HFO rates in *HFO area channels* decrease after surgery in the patients for whom postsurgical seizure freedom was achieved (Wilcoxon signed-rank: *p* = 0.005, z = 2.803) from 0.287 HFO/min (iqr 0.107) in the presurgical to 0.015 HFO/min (iqr 0.029) in the postsurgical EEG.

In the patients in whom postsurgical seizure freedom was achieved (Engel I), scalp HFO rates in *HFO area channels* were significantly decreased after surgery (Fig. 3), irrespective of their proximity to the skull defect resulting from craniotomy (Supplementary Table S1), whereas no significant change was established in the patients in whom postsurgical seizure freedom was not achieved. Furthermore, in patients in whom postsurgical seizure freedom was achieved, the median scalp HFO rate decreased from 0.287 HFO/min (iqr 0.107) in the presurgical EEG to 0.015 HFO/min (iqr 0.029) in the postsurgical EEG (Wilcoxon signed-rank, *p* = 0.005, z = 2.803). In contrast, in the patients in whom postsurgical seizure freedom was not achieved (Engel II-IV), the median scalp HFO rate did not differ significantly between the presurgical EEG (0.514 HFO/min, iqr 0.383) and the postsurgical EEG (0.506 HFO/min, iqr 0.461; Wilcoxon signed-rank, *p* = 0.465, z = 0.730).

### HFO rates near the skull defect do not increase after epilepsy surgery

**Figure 4:**
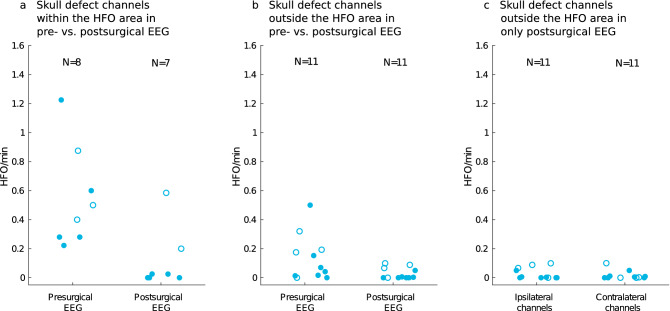
Scalp HFO rates of skull defect channels and their contralateral channels in presurgical and postsurgical EEG recordings. The patients for whom postsurgical seizure freedom was achieved (Engel I) are indicated by blue (full) circles, while patients with postsurgical seizure recurrence (Engel II–IV) are indicated by white (empty) circles. Of the 11 patients with *skull defect channels,* seven achieved postsurgical seizure freedom and four had postsurgical seizure recurrence. (**a**) Mean scalp HFO rates of *skull defect channels* within the HFO area significantly decreased (Wilcoxon signed-rank: *p* = 0.043, z = 2.023) from the presurgical (median 0.280 HFO/min, iqr 0.491) to the postsurgical EEG (median of 0.000 HFO/min, iqr 0.025) in the five patients for whom postsurgical seizure freedom was achieved. (**b**) Mean scalp HFO rates of *skull defect channels* outside the *HFO area* did not differ significantly (Wilcoxon signed-rank: *p* = 0.091, z = 1.690) between the presurgical (median 0.042 HFO/min, iqr 0.118) and postsurgical EEG (median 0.000 HFO/min, iqr 0.005) in the seven patients for whom postsurgical seizure freedom was achieved. (**c**) Mean HFO rates of *skull defect channels* outside the HFO area (median 0.003 HFO/min, iqr 0.063) did not differ significantly (Wilcoxon signed-rank: *p* = 0.726, z = 0.350) from their *contralateral channels* (median 0.003 HFO/min, iqr 0.011) in the postsurgical EEG of all patients, regardless of postsurgical seizure outcome.

In the patients in whom postsurgical seizure freedom was achieved (Engel I), mean HFO rates in *skull defect channels* within the HFO area decreased significantly (Wilcoxon signed-rank: *p* = 0.043, z = 2.023) from the presurgical (median 0.280 HFO/min, iqr 0.491) to the postsurgical EEG (median of 0.000 HFO/min, iqr 0.025) (Fig. 4a). In addition, mean HFO rates in *skull defect channels* outside the HFO area did not differ significantly (Wilcoxon signed-rank: *p* = 0.091, z = 1.690) between the presurgical (median 0.042 HFO/min, iqr 0.118) and the postsurgical EEG (median 0.000 HFO/min, iqr 0.005) (Fig. 4b). Finally, irrespective of the postsurgical seizure outcome, mean HFO rates in *skull defect channels* outside the HFO area (median 0.003 HFO/min, iqr 0.063) did not differ significantly (Wilcoxon signed-rank: *p* = 0.726, z = 0.350) from their *contralateral channels* (median 0.003 HFO/min, iqr 0.011) in the postsurgical EEG (Fig. 4c).

## Discussion

To our knowledge, the present study is the first to address the impact of skull defects resulting from craniotomy on scalp HFO, by evaluating alterations in HFO rates in relation to the presurgically defined HFO area, the resected area, and the postsurgical skull defect in presurgical and postsurgical EEG recordings from children and adolescents with drug-resistant focal lesional epilepsy. The results validate our earlier observation, deriving from a smaller exploratory cohort recorded with a custom-made amplifier, that HFO rates over the HFO area decrease after successful epilepsy surgery, thus reflecting treatment response, in a larger independent cohort recorded with a commercial device and followed-up considerably longer than our initial cohort^14^. In addition, in the current study we demonstrate for the first time that HFO rates both within and outside the HFO area remain largely unaffected by the skull defect resulting from craniotomy, thus excluding a confounding effect of the skull defect on HFO detection and confirming the validity of comparisons between consecutive EEG recordings. Our study thus serves to establish the utility of scalp HFO in evaluating treatment response, prognosticating seizure outcome, and dictating treatment management following pediatric epilepsy surgery.

### Scalp HFO rates decrease after successful epilepsy surgery

In our study, scalp HFO rates over the HFO area decreased following successful epilepsy surgery. These observations corroborate our previous findings, derived from an exploratory dataset recorded by a dedicated 8-channel low-noise amplifier^14^, connected to four adjacent electrodes over the presumed epileptogenic zone and to their contralateral electrodes, with an independent validation dataset recorded by a commercial device using the full 10–20 electrode array, without the requirement of a clear hypothesis regarding the localization of the epileptogenic zone. The previous dataset included a smaller cohort with a mean postsurgical follow-up of 1.2 years, while half of the patients had a follow-up of < 6 months. In contrast, the present results were derived from a larger cohort with a postsurgical follow-up of 2.2 years, while half of the patients had a follow-up of > 2 years. Our observations thus support the feasibility, applicability, and validity of our approach in a real-world pediatric setting, drawing from scalp EEG performed in clinical routine, and establish the potential of scalp HFO as a biomarker of treatment response in children and adolescents with focal lesional epilepsy undergoing epilepsy surgery.

Recent studies have supported a link between increased scalp HFO rates and the epileptogenic zone in focal lesional epilepsy in adult cohorts^15,16,18,34,35^, or between decreased scalp HFO rates and treatment response in various epilepsy syndromes^36–39^ in pediatric cohorts. However, data on the response to treatment of scalp HFO related to a focal lesion in pediatric cohorts is scarce, limited to a single study that followed the evolution of HFO rates over consecutive EEG recordings before, during, and after epilepsy surgery^14^. In addition to establishing a link between HFO in the intraoperative ECoG and HFO in the scalp EEG, that study^14^ showed for the first time that the resection of the cortical HFO generators results in a reduction of HFO rates recorded in scalp EEG, paving the way for our current work. However, these preliminary observations were derived from only eight patients with consecutive presurgical and postsurgical scalp EEG recordings, of whom only half had a postsurgical follow-up of over a year. In the present study, the decrease of scalp HFO rates at the 3-month follow-up corresponded to seizure freedom 2.5 years after surgery and after ASM withdrawal in over half of patients, thus supporting the validity and generalizability of our results. Scalp HFO rates also dropped with decreasing seizure frequency in our pediatric focal lesional epilepsy cohort, thus reflecting treatment response.

### Scalp HFO rates are not impacted by the skull defect resulting from craniotomy

Scalp HFO rates both within and outside the HFO area remained largely unaffected by the skull incision resulting from craniotomy, thus excluding a confounding effect of the skull defect on HFO detection and confirming the validity of comparisons between consecutive presurgical and postsurgical EEG recordings, as performed both in our previous, exploratory study^14^, and in our current, validating dataset. Furthermore, while scalp HFO rates in channels over the HFO area decreased following successful epilepsy surgery, irrespective of their proximity to the skull defect, scalp HFO rates in channels outside the HFO area but close to the skull defect did not increase following surgery. Our findings suggest that monitoring disease severity and response to treatment after craniotomy for epilepsy surgery is feasible and reliable since the resulting skull defect does not interfere with HFO detection.

In the present study, we have shown for the first time that scalp HFO rates are not impacted by the skull defect resulting from craniotomy, including electrode contacts within close distance from the skull defect. This very reassuring finding concerning HFO in scalp EEG contrasts with several reports of skull defects modifying the appearance of spikes in scalp EEG to the extent that they can be mistaken for electrode artifacts and thus escape detection^40,41^. In these reports, the scalp EEG recorded by a standard 10–20 electrode array illustrated cortically generated spikes as phase reversals without a physiological field due to the compact field configuration resulting from the skull defect. Furthermore, mu and beta rhythms may resemble spikes in the context of a pronounced breach rhythm^42^, thus adding to the risk of false positives in spike detection at the presence of a skull defect.

Beyond the detection and interpretation of scalp EEG signals in the HFO range in follow-up recordings after epilepsy surgery, as implemented in our study, our observations have crucial implications for patients undergoing presurgical investigation after failed epilepsy surgery or after a first resection guided by surgical indications, as in tumors and cavernomas. Considering that reoperations in pediatric epilepsy surgery are no rarity^2,3,5,43–45^, the fact that scalp HFO rates remain unaffected by the skull defect resulting from craniotomy is particularly encouraging for the use of scalp HFO both in the postsurgical setting, to guide crucial decisions such as the initiation of ASM withdrawal and in the presurgical setting, to guide the decision for epilepsy surgery and the delineation of surgical resection in selected candidates.

### Future directions

Beyond the specific setting of epilepsy surgery, the correlation between HFO rates and seizure frequency may provide a valuable tool for monitoring disease severity and treatment success in this patient group, irrespective of the therapeutic approach. Novel and reliable non-invasive EEG biomarkers are urgently needed, particularly in pediatric epilepsy, since currently available biomarkers are insufficient to support treatment management. Spikes, the EEG biomarker currently in clinical use, lack specificity as they are sometimes recorded even in the absence of epilepsy^46,47^, while their rates correlate neither with seizure frequency nor with treatment response in the presence of epilepsy^48^. Considering that epilepsy incidence is higher in early life^49^ and pediatric epilepsies often take a catastrophic course^2,43,50^, novel biomarkers are likely to have the most significant impact in children and adolescents. A novel epilepsy biomarker that would efficiently monitor the disease state, thus guiding treatment choices, is poised to drastically change epilepsy management, possibly improving not only seizure but also cognitive outcomes in this vulnerable age group.

Despite the encouraging results of our study, scalp HFO need to be evaluated as a novel method against the current standards of prognostication in sufficiently large samples from multicentric studies. Furthermore, the validity and reliability of scalp HFO as an independent prognostic factor have to be assessed in multifactorial clinical prognosis scores allowing the integration of clinical, EEG, and imaging data and scalp HFO rates. In particular, conclusive data regarding the seizure recurrence risk after ASM withdrawal are required and the identification of relevant risk factors will have to outperform currently available predictive models^51^ with their inherent accuracy issues^52^ by integrating more informative diagnostic tools as covariates, such as neurophysiological markers of the epileptogenic network, including scalp HFO.

Finally, beyond the effect of skull defects resulting from craniotomy on the validity of postsurgical scalp HFO, as investigated in the present study, further factors should be addressed that may potentially affect postsurgical scalp HFO, such as the extent of craniotomy or the extent of resection. Although a larger craniotomy and resection may have a greater overall impact on scalp EEG signal, scalp HFOs have been shown to be generated by and synchronized over small brain regions^18^, thus adding to the ambiguity concerning the impact of craniotomy and resection extent on scalp HFO. This direction could be pursued in future datasets of pre- and postsurgical scalp EEG recordings from larger pediatric cohorts with drug-resistant focal lesional epilepsy undergoing resective epilepsy surgery that will serve to validate and expand the findings of the current study. In addition, larger datasets than the one analyzed in our study could potentially serve to develop statistically more robust modelling approaches, e.g. taking into account the correlation of within-subject data (multiple HFO rates and electrode distance measurements over time per channel within a given subject), thus refining our current conclusions.

## Conclusion

We demonstrated that the decrease of scalp HFO rates over the HFO area correlated with long-term seizure freedom in our patients, even after the ASM had been withdrawn. In parallel, scalp HFO rates both within and outside the HFO area remained unaffected by the skull defect resulting from craniotomy, even in electrodes within close distance from the skull defect. Our study thus establishes the potential of scalp HFO for assessing treatment response in the pediatric population undergoing epilepsy surgery, potentially facilitating seizure outcome prognostication and thus guiding crucial therapeutic decisions, such as the initiation of ASM withdrawal.

## Supplementary Information

Supplementary Information.

## Data Availability

The raw EEG data and the HFO markings will be published upon acceptance of the article.

## Code availability

The HFO detection software is freely available at the GitHub repository (https://github.com/ZurichNCH/Automatic-High-Frequency-Oscillation-Detector).
