# Supplementary material for: Scalp HFO rates decrease after successful epilepsy surgery and are not impacted by the skull defect resulting from craniotomy
Source: Sci Rep. 2022 Jan 25;12:1301. doi: 10.1038/s41598-022-05373-x (PMC8789862; doi:10.1038/s41598-022-05373-x)
Supplement: Supplementary file 1 — Supplementary Information. [file 41598_2022_5373_MOESM1_ESM.pdf]

SCALP HFO RATES DECREASE AFTER SUCCESSFUL EPILEPSY SURGERY AND ARE NOT IMPACTED BY THE SKULL DEFECT RESULTING FROM CRANIOTOMY

Dorottya Cserpan[1], Antonio Gennari[1,2], Luca Gaito[1,3], Santo Pietro Lo Biundo[1], Ruth Tuura[2,4,5], Johannes Sarnthein[3,4,6], Georgia Ramantani[1,4,5,+]

[1] Department of Neuropediatrics, University Children's Hospital Zurich, Switzerland

[2] MR-Research Centre, University Children's Hospital Zurich, Switzerland

[3] Department of Neurosurgery, University Hospital Zurich, Switzerland

[4] University of Zurich, Switzerland

[5] Children’s Research Centre, University Children's Hospital Zurich, Switzerland

[6] Klinisches Neurozentrum Zürich, University Hospital Zurich, Switzerland

[+]Corresponding author: georgia.ramantani@kispi.uzh.ch

Georgia Ramantani, MD, Ph.D.; Neuropediatrics, University Children's Hospital Zurich, Steinwiesstrasse 75, 8032 Zurich, Switzerland; phone: +41 44 266 75 92

Supplementary Information

Patient Nr.	SEIZURE OUTCOME (Engel)	HFO AREA CHANNELS		SKULL DEFECT CHANNELS OUTSIDE THE HFO AREA		CONTRALATERAL SKULL DEFECT CHANNELS
		Presurgical HFO rate	Postsurgical HFO rate	Presurgical HFO rate	Postsurgical HFO rate	Postsurgical HFO rate
1	IV	0.578	0.125	0.320	0.000	0.000
2	I	0.267	0.000	0.017	0.000	0.000
3	IV	0.450	0.420	0.175	0.067	0.100
4	I	0.226	0.015	0.042	0.003	0.005
5	I	1.388	0.000	0.500	0.000	0.008
6	IV	0.400	0.591	0.000	0.099	0.003
7	I	0.422	0.067	0.152	0.006	0.011
8	I	0.293	0.000	0.013	0.000	0.000
9	I	0.333	0.048	-	-	-
10	IV	1.038	0.878	0.193	0.088	0.000
11	I	0.300	0.030	-	-	-
12	I	0.280	0.025	0.070	0.000	0.050
13	I	0.040	0.006	-	-	-
14	I	0.124	0.014	0.000	0.050	0.000

Supplementary Table 1. HFO rates in the channels of interest in presurgical and postsurgical scalp EEG recordings. HFO rates are given as the mean of the HFO area channels, the skull defect channels, and their contralateral channels, in HFO/min.

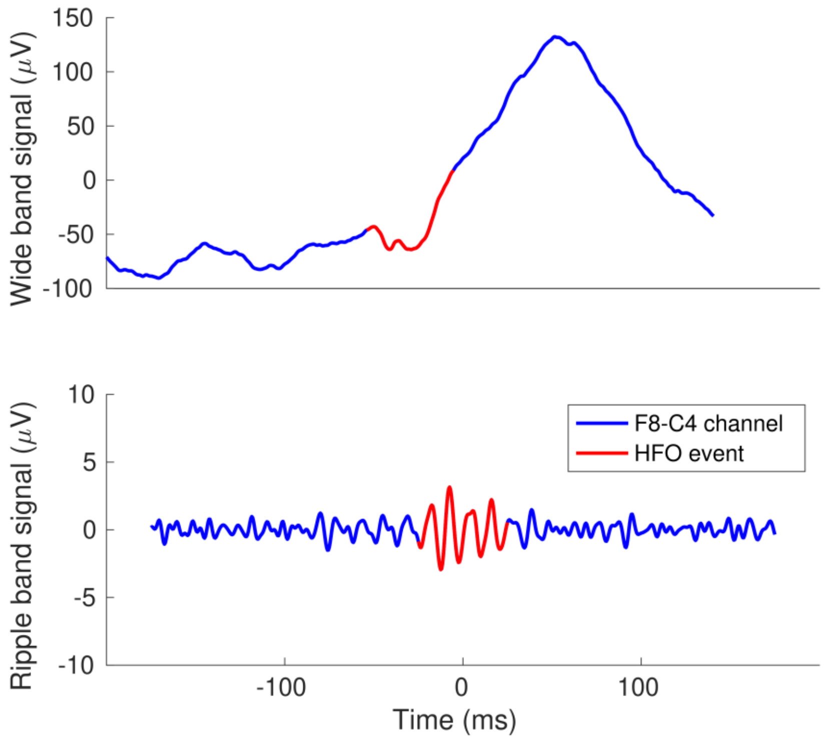

Supplementary Figure 1. An exemplary HFO event is shown in red in the wide band (upper panel) and ripple band (lower panel) signal, as detected on the T6-C4 channel in the scalp EEG recording of Patient 5.

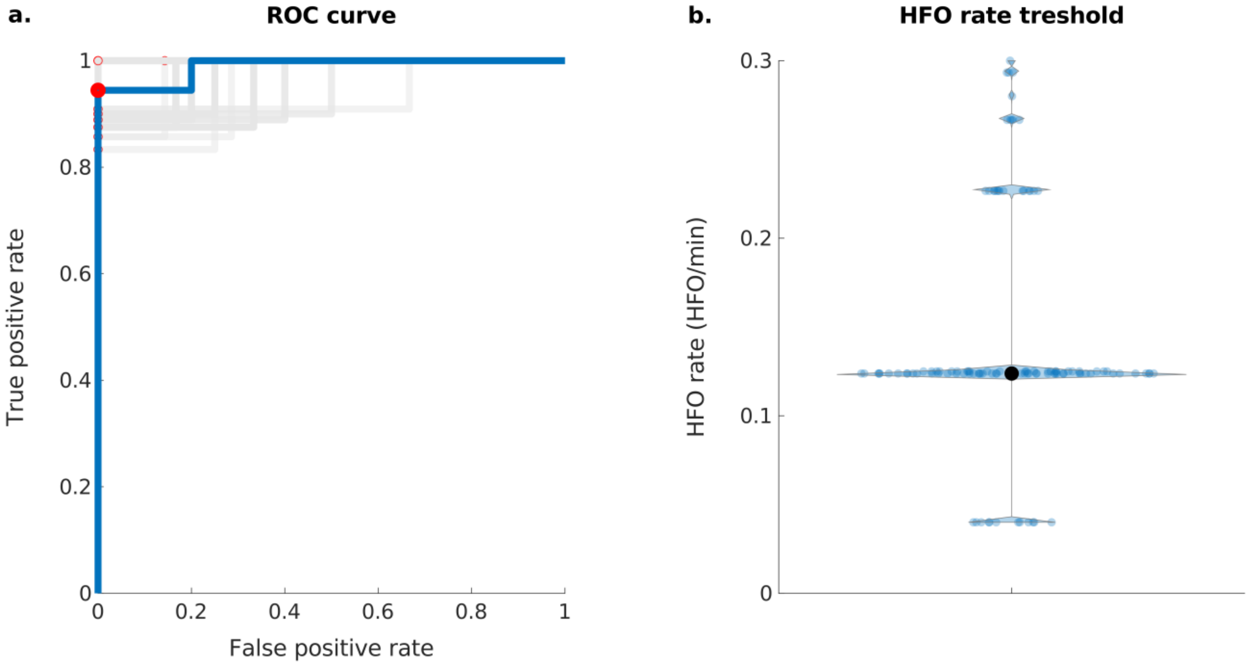

Supplementary Figure 2. a) Receiver Operating Characteristic **(**ROC) curve, constructed by plotting the true positive rate on the y axis against the false positive rate on the x axis, based on all recordings (outlined in blue) and based on recordings split into training- and test datasets (outlined in grey). The ROC curve illustrates the classification of our datasets based on the HFO rate threshold of the optimal operating point. The HFO rate threshold estimated by the ROC curve based on all recordings was 0.124 HFO/min. In this case, scalp HFO rates in *HFO area channels* exceeded the 0.124 HFO/min threshold in 17 recordings, all from patients with active epilepsy (PPV = 100%) and fell below this threshold in 11 recordings, ten from patients that achieved postsurgical seizure freedom (NPV = 91%, accuracy = 96%). In addition, we challenged the predictive performance of the HFO threshold rate methodology by applying two-fold cross-validation. We randomly shuffled the data array and split it into two equal size datasets d_0 and d_1, each containing 14 scalp EEG recordings. We then trained on d_0 and validated on d_1, followed by training on d_1 and validating on d_0, then repeating this procedure two times the number of recordings. The two-fold cross-validation supported the validity of our findings (median PPV = 100%, iqr 0%; median NPV = 83%, iqr 29%; median accuracy = 93%, iqr 7%), while the median HFO rate threshold matched the threshold deriving from the full dataset (median 0.124, iqr 0.001).
